# EB1 phosphorylation mediates the functions of ASK1 in pancreatic cancer development

**DOI:** 10.18632/oncotarget.21004

**Published:** 2017-09-18

**Authors:** Siqi Gao, Youguang Luo, Xiaofan Wu, Yuanyuan Li, Yunqiang Zhou, Rui Lyu, Min Liu, Dengwen Li, Jun Zhou

**Affiliations:** ^1^ State Key Laboratory of Medicinal Chemical Biology, Key Laboratory of Bioactive Materials of the Ministry of Education, College of Life Sciences, Nankai University, Tianjin 300071, China; ^2^ Shandong Provincial Key Laboratory of Animal Resistance Biology, Institute of Biomedical Sciences, College of Life Sciences, Shandong Normal University, Jinan, Shandong 250014, China

**Keywords:** EB1, phosphorylation, ASK1, pancreatic cancer

## Abstract

Pancreatic cancer has a poor prognosis due to its rapid rate of metastasis and frequent late-stage diagnosis. An improved understanding of the molecular mechanisms underlying this disease is urgently needed to promote the development of improved diagnostic tools and more effective therapies. Apoptosis signal-regulating kinase 1 (ASK1) has been shown to be overexpressed in pancreatic cancer and to promote the proliferation of pancreatic cancer cells in a kinase activity-dependent manner. However, the molecular mechanisms by which ASK1 promotes cell proliferation remain to be elucidated. In this study, we report that the phosphorylation of end-binding protein 1 (EB1) at threonine 206 (pT206-EB1), which is catalyzed by ASK1, is increased in pancreatic cancer tissues. We further find that the level of pT206-EB1 correlates with that of ASK1 in cancer tissues. Additionally, ASK1 localizes to spindle poles, and knockdown of ASK1 results in the formation of multipolar spindles. Moreover, we show that depletion of ASK1 or disruption of EB1 phosphorylation inhibits spindle microtubule dynamics in pancreatic cancer cells. Collectively, these findings suggest that EB1 phosphorylation mediates the functions of ASK1 in pancreatic cancer development.

## INTRODUCTION

Pancreatic cancer has a very poor prognosis due to its rapid progression and lack of early detection. Less than six percent of patients with pancreatic cancer survive more than five years, primarily due to the advanced stage of the disease at diagnosis [1, 2]. Mutations in a subset of genes, including *KRAS*, *CDKN2A*, *TP53*, and *SMAD4*, have been found to be associated with pancreatic cancer initiation and development [3]. However, a more comprehensive understanding of the molecular events that occur during early pathogenesis is needed to improve prognosis.

ASK1 is a ubiquitously expressed serine/threonine mitogen-associated protein kinase kinase kinase (MAP3K) that activates the c-Jun N-terminal kinase (JNK) and p38 signaling pathways [4–6]. Aberrant expression or mutation of ASK1 has been implicated in the pathogenesis of cardiovascular and neurodegenerative diseases, diabetes, and cancer [7]. Recently, ASK1 overexpression has been shown to play a crucial role in pancreatic cancer development through the promotion of cell proliferation [8]. This oncogenic role has been shown to require the kinase activity of ASK1, but the downstream effectors in the process remain elusive.

A known target of ASK1 is end-binding protein 1 (EB1), a key member of microtubule plus end-tracking proteins (+TIPs) that regulates the dynamic properties of microtubules and microtubule-mediated cellular processes [9–13]. In addition, EB1 recruits other members of +TIPs to microtubule plus ends to regulate microtubule dynamics and functions [14–17]. Recently, ASK1-mediated EB1 phosphorylation has been demonstrated to modulate the orientation and positioning of the mitotic spindle, a critical structure required for the balanced distribution of chromosomes and cellular contents between daughter cells [18–22]. However, it is unknown whether EB1 phosphorylation is involved in the functions of ASK1 in pancreatic cancer progression.

## RESULTS

### EB1 phosphorylation correlates with ASK1 expression in pancreatic cancer

**Figure 1:**
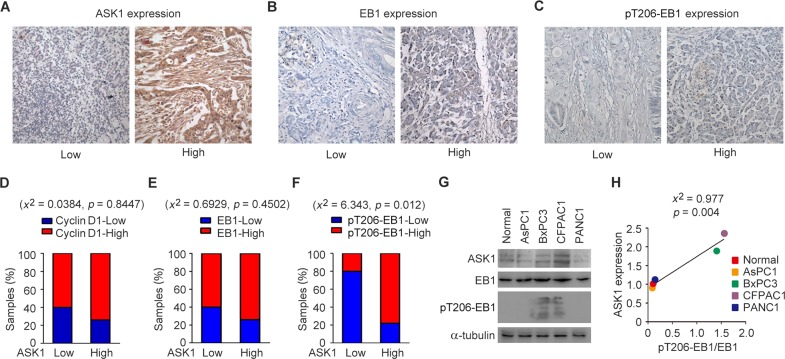
EB1 phosphorylation correlates with ASK1 expression in pancreatic cancer **(A-C)** Representative images showing immunohistochemical staining of ASK1, EB1, and pT206-EB1 expression in pancreatic cancer tissue samples. **(D-F)** Quantification of cyclin D1 (D), EB1 (E) and pT206-EB1 (F) expression in pancreatic cancer tissues (n = 19) with high or low levels of ASK1 expression. Chi-square (*x*^2^) tests were used to determine the correlation. **(G)** Western blot analysis of ASK1, EB1, pT206-EB1, and α-tubulin expression in normal pancreatic epithelial cells and pancreatic cancer cell lines. **(H)** Experiments were performed as in G, and the correlation between ASK1 expression and EB1 phosphorylation (the level of pT206-EB1 divided by the level of EB1) was determined by *x*^2^ tests. Error bars represent mean ± standard error of the mean (SEM).

To understand whether ASK1-mediated phosphorylation of EB1 contributes to pancreatic cancer development, we analyzed the expression of ASK1, EB1, and EB1 phosphorylated at T206 (pT206-EB1) in clinical samples from pancreatic cancer patients (Figures 1A-1C). Each pancreatic tissue sample was classified as having low or high ASK1 expression. As previously described, we found that more than 65% of tissues that had high ASK1 expression also expressed high levels of cyclin D1 (Figure 1D) [8]. Intriguingly, more than 65% of samples that had high EB1 expression (Figure 1E) and 75% of tissues with high levels of pT206-EB1 also exhibited high ASK1 expression (Figure 1F). To further explore this relationship, we analyzed the expression of EB1 and pT206-EB1 in normal pancreatic epithelial cells and in four pancreatic cancer cell lines: AsPC1, BxPC3, CFPAC1, and PANC1. Immunoblot analysis revealed that expression of pT206-EB1 was higher in the BxPC3 and CFPAC1 cell lines, which also had high ASK1 expression (Figure 1G). Chi-square (*x*^2^) test further showed that the level of ASK1 expression significantly correlated with the level of EB1 phosphorylation in these cells (Figure 1H). Together, these data suggest that ASK1-mediated phosphorylation of EB1 may be a regulatory mechanism that contributes to pancreatic cancer cell proliferation.

### Depletion of spindle pole-localized ASK1 results in the formation of multipolar spindles

**Figure 2:**
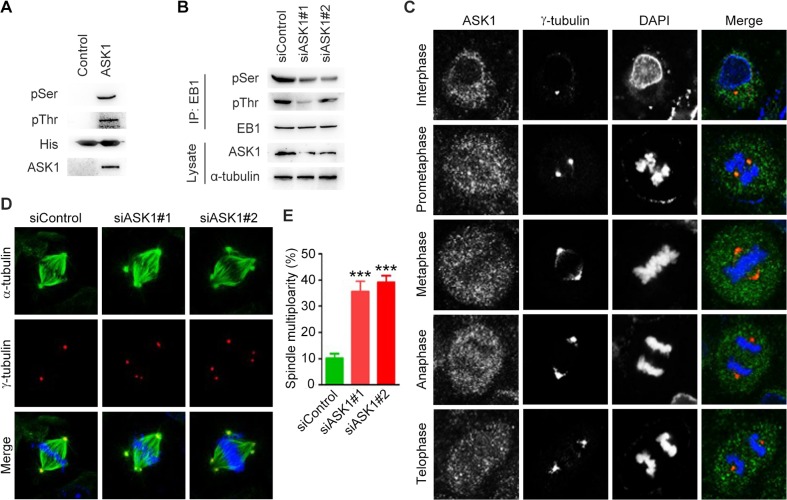
Depletion of spindle pole-localized ASK1 leads to the formation of multipolar spindles **(A)** *In vitro* kinase assays were performed by using ASK1 immunoprecipitate with bacterially purified His-EB1 as a substrate. The reaction mixture was then subjected to immunoblotting with phosphoserine (pSer) and phosphothreonine (pThr) antibodies. **(B)** PANC1 cells transfected with control or ASK1 siRNAs were lysed, and immunoprecipitation and immunblotting were then performed with the indicated antibodies. **(C)** PANC1 cells were stained with ASK1 and α-tubulin antibodies. Representative images of cells in interphase and various phases of mitosis. **(D)** PANC1 cells transfected with control or ASK1 siRNAs were stained with α-tubulin and γ-tubulin antibodies. **(E)** Quantification of cells with multipolar spindles. Student's t-tests were performed. ^***^, *p* < 0.001. Error bars represent mean ± SEM.

We then analyzed whether EB1 undergoes phosphorylation by ASK1 in pancreatic cancer cells. We performed *in vitro* kinase assays using ASK1 immunoprecipitate and bacterially purified His-EB1. Immunoblotting of the reaction mixture with phosphoserine and phosphothreonine antibodies revealed that EB1 was phosphorylated at both serine and threonine residues by ASK1 (Figure 2A). Furthermore, siRNA-mediated knockdown of ASK1 expression in PANC1 cells significantly decreased serine/threonine phosphorylation of EB1 (Figure 2B). In this study, we chose PANC1 cells for further experiments, because of the high efficiency of the ASK1 siRNAs in decreasing ASK1 expression and EB1 phosphorylation in this cell line.

EB1 is a key regulator of the dynamic microtubule behavior required for the function of spindle microtubules during mitosis [14]. To analyze the role of ASK1 in the regulation of spindle microtubules, we first assessed the localization of ASK1 in PANC1 cells during mitotic progression. Immunofluorescence analysis showed that ASK1 localized to the spindle poles. This localization was especially pronounced during prometaphase (Figure 2C), suggesting a role for ASK1 in the regulation of the mitotic spindle. We next compared spindle microtubules in control PANC1 cells and in cells treated with ASK1-targeted siRNAs (Figure 2D) and found an increase in the number of cells with multipolar spindles in ASK1-depleted cells (Figure 2E). These results provide evidence supporting a role for ASK1 in spindle integrity, a function consistent with the known role of EB1.

### Depletion of ASK1 impairs microtubule dynamics

**Figure 3:**
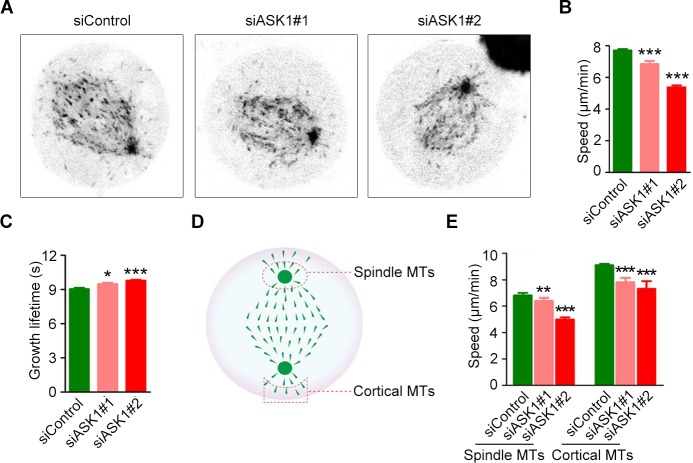
ASK1 depletion decreases microtubule dynamics **(A)** Cells were transfected with control or ASK1-targeted siRNAs and GFP-EB1. Time-lapse images of GFP-EB1 were captured at 2-sec intervals. **(B-C)** Microtubule growth tracks were analyzed with PlusTipTracker software; Microtubule growth speed (B) and lifetime (C) were measured. **(D)** Schematic depicting the method for measuring spindle and cortical microtubule dynamics. **(E)** Graphical representation of the speed of spindle or cortical microtubule dynamics in cells transfected with control or ASK1-targeted siRNAs. Student's t-tests were performed. ^*^, *p* < 0.05; ^**^, *p* < 0.01; ^***^, *p* < 0.001. Error bars represent mean ± SEM.

To analyze the role of ASK1 in spindle integrity, we transfected cells with GFP-EB1, which binds microtubule plus ends and can be used to track microtubule growth dynamics (Figure 3A). We found that in cells depleted of ASK1, microtubules showed decreased dynamics, with lower growth speeds (Figure 3B) and longer growth times (Figure 3C) compared to microtubules in control cells. Next, we compared the dynamics of spindle and cortical microtubules (Figure 3D). ASK1 depletion in cells resulted in a decrease in both spindle and cortical microtubule growth speed (Figure 3E), indicating that ASK1 may regulate spindle stability and polarity by influencing spindle microtubule dynamics.

### Overexpression of phosphorylation-deficient EB1 affects microtubule dynamics

**Figure 4:**
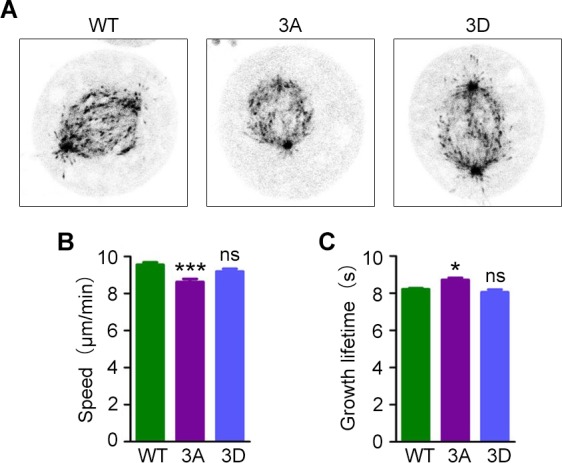
Expression of a phosphorylation-deficient EB1 mutant decreases microtubule dynamics **(A)** Cells were transfected with GFP-EB1, GFP-EB1 3A or GFP-EB1 3D, and time-lapse images of GFP-EB1 comets were captured at 2-sec intervals. **(B-C)** Microtubule growth tracks were analyzed with PlusTipTracker software, and microtubule growth speed (B) and lifetime (C) were measured. Student's t-tests were performed. ^*^, *p* < 0.05; ^***^, *p* < 0.001; ns, not significant. Error bars represent mean ± SEM.

To assess the role of EB1 phosphorylation in the regulation of microtubule dynamics, we expressed GFP-EB1; a phosphorylation-deficient GFP-EB1 mutant, in which T206 and other two phosphosites (S40 and T154) were mutated to alanine (3A); or a phosphorylation mimic GFP-EB1 mutant, in which the three residues were mutated to aspartic acid (3D) [18]. We then tracked microtubule plus tips using live imaging to analyze microtubule dynamics (Figure 4A). We found that in the phosphorylation-deficient 3A mutant, comets had slower growth speeds and longer growth times, while microtubule growth was unaffected in cells expressing the phosphorylation-mimic 3D mutant (Figure 4B and 4C). Thus, loss of EB1 phosphorylation has effects on spindle microtubule dynamics that are similar to the results of ASK1 depletion, suggesting that ASK1 regulates spindle microtubule dynamics through phosphorylation of EB1. Furthermore, S40, T154, and T206 may be critical phosphorylation sites in EB1-mediated microtubule regulation.

### ASK1 depletion does not affect cortical actin or cell shape

**Figure 5:**
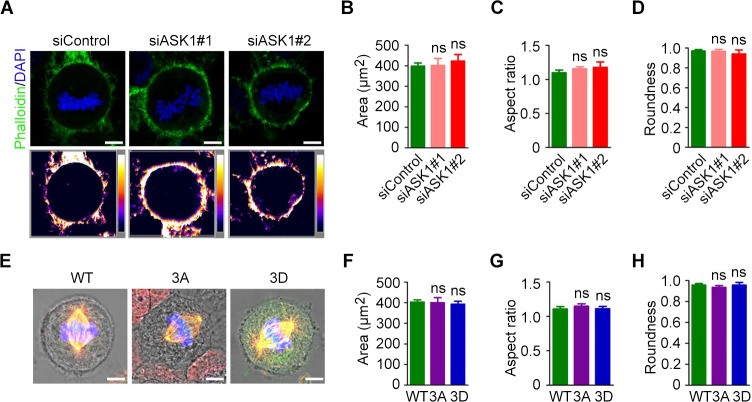
Cortical actin and cell shape are not affected by ASK1 depletion **(A)** Cells were transfected with control or ASK1-targeted siRNAs and stained with phalloidin and DAPI. Intensity maps of phalloidin staining are shown. Area **(B)**, aspect ratio **(C)**, and roundness **(D)** of mitotic cells were measured with Image J software. **(E)** Cells were transfected with GFP-EB1, GFP-EB1 3A, or GFP-EB1 3D and immunostained with α-tubulin antibodies; representative immunofluorescence/phase-contrast images are shown. Area **(F)**, aspect ratio **(G)**, and roundness **(H)** of mitotic cells were measured using Image J software. Student's t-tests were performed. ns, not significant. Error bars represent mean ± SEM.

Because spindle microtubules also play a role in maintaining cell shape in mitotic cells [19], we sought to determine the role of ASK1 in cell morphology. Cells were treated with control siRNAs or siRNAs targeting ASK1 and stained with phalloidin to generate intensity maps (Figure 5A). Next, we measured the area (Figure 5B), aspect ratio (Figure 5C), and roundness (Figure 5D) of cells using Image J software; we found no significant difference between control and ASK1-depleted cells. Additionally, GFP-EB1, GFP-EB1 3A, and GFP-EB1 3D mutants were expressed in cells, and phase-contrast images were captured to visualize cell edges (Figure 5E). The area (Figure 5F), aspect ratio (Figure 5G), and roundness (Figure 5H) were measured, revealing no obvious differences in these parameters when compared to control cells. These results indicate that, although ASK1 affects spindle microtubule dynamics and polarity, ASK1 does not affect the shape of mitotic cells.

## DISCUSSION

Pancreatic cancer is a highly malignant disease and a leading cause of cancer-associated deaths. However, the underlying mechanisms that drive pancreatic cancer remain largely unknown. ASK1 has been shown previously to play an important role in pancreatic cancer cell proliferation [8], but how ASK1 exerts this effect remains to be elucidated. In this study, our data demonstrate that EB1 phosphorylation mediates the functions of ASK1 in pancreatic cancer development. Specifically, we show that ASK1 phosphorylates EB1 to regulate spindle dynamics and stability. EB1 is known to have a crucial role in the regulation of microtubule dynamics, and its dysregulation has been implicated in cancer development. In lung cancer, EB1 inhibits cell death by inducing ROS-mediated, NF-κB-dependent Bax signaling cascades [23]. EB1 also shows a high expression in breast cancer and promotes breast cancer cell proliferation, which has a strong correlation with Aurora-B activity [24]. Our findings reveal a novel function of EB1 to promote pancreatic cancer development through altering spindle dynamics and stability. Dysregulated EB1 phosphorylation in these processes may promote pancreatic cancer cell proliferation.

ASK1 has been reported previously to play diverse roles in other types of cancers. For example, in skin cancer, ASK1 stimulates cytokine secretion to promote the proliferation of cancer cells [6]. In addition, in liver cancer, ASK1 regulates cancer development by promoting apoptosis and enhancing the expression of p21 [25]. Our data show that ASK1 affects microtubule dynamics, suggesting a novel mechanism by which ASK1 contributes to the progression of pancreatic cancer. Our data also reveal that ASK1 knockdown and EB1 mutants have no obvious effect on mitotic cell roundness. The surface tension of the actin cortex is known to increase rapidly in mitotic cells [26, 27]. Although spindle microtubules are also involved in maintaining cell roundness [19], our results suggest that the alteration of microtubule dynamics may be not sufficient for dramatically changing cell morphology.

We have demonstrated previously that ASK1 regulates mitotic spindle positioning and orientation [18]. In agreement with these findings, the present study shows that ASK1 also promotes spindle microtubule dynamics. These changes in microtubule dynamics may be the underlying cause of the previously reported defects in spindle positioning and orientation. Inhibitors of ASK1 kinase activity are in development for the treatment of several diseases, including gastric cancer [28], neurodegenerative disorders [29], and ischemia-reperfusion injury [30]. In this study, we provide evidence that ASK1 and its substrate, EB1, contribute to pancreatic cancer development, suggesting that these inhibitors could have therapeutic value in this setting.

## MATERIALS AND METHODS

### Antibodies, chemicals, siRNAs and plasmids

Primary antibodies directed against ASK1 (Abcam), EB1 (BD Biosciences), α-tubulin (Abcam), γ-tubulin (Abcam), phosphoserine (Cell Signaling Technology), phosphothreonine (Cell Signaling Technology), and horseradish peroxidase-conjugated secondary antibodies (Santa Cruz), rhodamine- or fluorescein-conjugated secondary antibodies (Jackson ImmunoResearch) were obtained from the indicated sources. The pT206-EB1 customized antibody was obtained from GL. DAPI, and fluorescein-conjugated phalloidin were purchased from Sigma-Aldrich. The control siRNA (5′-CGUACGCGGAAUACUUCGA-3′) and ASK1 siRNAs (#1: 5′-GCACUCCU-UCAUCGAGCU-3′; #2: 5′-GGUAUACAUGAGUGGAAUU-3′) were synthesized by Ribo Bio. Mammalian expression plasmids for GFP-EB1 were constructed by PCR using the pEGFP-N1 vector as described previously [31].

### Cell culture and transfection

Human pancreatic cancer cell lines were purchased from the American Type Culture Collection. AsPC1 and BxPC3 cells were cultured in RPMI-1640 medium supplemented with 10% fetal bovine serum (FBS). PANC1 cells were cultured in Dulbecco's Modified Eagle's Medium supplemented with 10% FBS. CFPAC1 cells were cultured in Iscove's Modified Dulbecco's Medium supplemented with 10% FBS. Cells were maintained in a humidified incubator containing 5% CO_2_ at 37°C. Plasmids and siRNAs were transfected using TurboFect (Thermo Fisher Scientific) or Lipofectamine RNAiMAX (Invitrogen), respectively [32, 33].

### Immunohistochemistry

Human pancreatic cancer tissues were obtained from patients who underwent surgical resection at Shanxian Dongda Hospital. Paraffin-embedded tissues were sliced into 5-μm sections, deparaffinized, and rehydrated with xylene and graded alcohols. Endogenous peroxidase activity was inactivated, and antigen retrieval was performed [34]. Subsequently, the sections were blocked with 2% bovine serum albumin, incubated with the primary antibody, followed by incubation with a biotinylated secondary antibody and streptavidin-biotin-peroxidase. Diaminobenzidine was used as a chromogenic substrate, followed by counterstaining with hematoxylin. The level of protein expression was classified as described previously [35, 36].

### Immunoblotting and immunoprecipitation

Protein samples were separated using SDS-PAGE and transferred to polyvinylidene difluoride membranes (Millipore). Membranes were subsequently blocked with 5% fat-free milk and incubated with primary antibodies (1:2000), followed by horseradish peroxidase-conjugated secondary antibodies [37–39]. Bound antibodies were visualized with enhanced chemiluminescence detection reagent (Millipore). For immunoprecipitation, cell lysates were incubated with antibody-coated agarose beads at 4°C for 4 h. The beads were washed, boiled in the SDS loading buffer, and then examined by immunoblotting [40–42].

### *In vitro* kinase assay

*In vitro* kinase assays were performed at 30°C for 2.5 h in the kinase reaction buffer (Cell Signaling Technology), by using ASK1 immunoprecipitate and bacterially purified His-EB1 as described previously [18].

### Immunofluorescence microscopy

Cells were grown on glass coverslips and fixed with ice-cold methanol for 3 minutes. Fixed cells were blocked with 2% bovine serum albumin and incubated with primary antibodies, followed by incubation with rhodamine- or fluorescein-conjugated secondary antibodies [43, 44]. The cells were then stained with phalloidin and DAPI [45]. The coverslips were then mounted onto slides and imaged using a confocal microscope (Zeiss).

### Live-cell imaging and analysis

Cells transfected with GFP-EB1 or GFP-EB1 mutants were imaged with a confocal microscope (Leica) at 37°C in at atmosphere containing 5% CO_2_. Time-lapse images of GFP-EB1 comets were captured at 2-sec intervals for 2 min as described previously [46]. Microtubule dynamics were analyzed using PlusTipTracker software [47, 48].

### Statistics

Student's t-tests were performed for significant differences. Chi-square (*x*^2^) tests were performed to determine the correlation between two samples.
